# Right ventricular outflow tract tachycardia worsened during pregnancy

**DOI:** 10.11604/pamj.2015.20.60.5864

**Published:** 2015-01-22

**Authors:** Yibar Kambiré, Lassina Konaté, Georges Rosario Christian Millogo, Elodie Sib, Myriam Amoussou, Lucie Valérie Adélaïde Nebié, Ali Niakara

**Affiliations:** 1 Department of Medicine and Specialties, National Hospital Blaise Compaoré, 11 PoBox 104 Ouagadougou, CMS 11, Burkina Faso; 2 Cardiology Department, Teaching Hospital Yalgado Ouedraogo, Ouagadougou, Burkina Faso; 3 International Polyclinic of Ouagadougou, 01 BP 2092 Ouagadougou 01, Burkina Faso; 4 Mother and Child Department, National Hospital Blaise Comparore, 11 PoBox 104 Ouagadougou, CMS 11, Burkina Faso

**Keywords:** Ventricular tachycardia, pregnancy, radiofrequency, Africa

## Abstract

We report the case of a 35 years old woman without underlying heart disease who was diagnosed with a right ventricular outflow tract tachycardia worsened during pregnancy. The diagnosis of ventricular tachycardia was made early in her pregnancy course but the patient had symptoms three months earlier. Her disease course was marked by rhythmic storms during the second trimester of pregnancy that led to three hospitalizations accounting for about two weeks in total. The combination of nadolol 80 mg and flecainide tablets 150 mg improved her rhythmic storms. Radiofrequency allowed a radical cure of this ventricular tachycardia. The patient is now asymptomatic 27 months after radiofrequency treatment.

## Introduction

Cardiac arrhythmia can occur during pregnancy in the presence or absence of an underlying organic heart disease. They are most often made of ventricular or supraventricular extrasystoles with a good prognosis. Sometime, they need treatment that can be difficult. We report the case of right ventricular outflow tract tachycardia worsened in pregnancy.

## Patient and observation

Mrs. AMS, 35 years old is seen at her outpatient visit on October 3, 2011 for a three months history of paroxysmal lipothymia occurring at rest, including while in bed and while driving her car. The episodes of lipothymia are accompanied by dizziness, light-headedness and short lasting blurred vision, all preceded by palpitations. There is neither dyspnea nor stress-related symptoms. Her cardiovascular risk factors are her overweight (BMI = 29.14) and an early death of her father at age 33 years due to kidney failure associated with hypertension. Her past medical history reveals asthma attacks that were treated as they occurred. Her last asthma attack happened over a year ago. She is primipara with a healthy child. The first episodes of palpitations occurred in July 2011 with a normal electrocardiogram. She was not pregnant then.

Cardiovascular examination is normal with a heart rate of 84 bpm, no heart murmur nor signs of heart failure. Blood pressure while lying down and standing up is 120/70 mmHg on her right arm and 110/60 mmHg on her left. Examination of other organs is normal. The 12-lead surface ECG is normal with regular sinus rhythm at 81 bpm and a normal adjusted QT interval. Doppler echocardiography is normal. Systolic function of both ventricles was preserved (LVEF = 70%; TAPSE = 23 mm). For instance, there is no left or right ventricular hypertrophy or dilation. 24-hoursHolter ECG shows a sinus rhythm at 88 bpm without rhythm or conduction disorders. Her electrolytes, creatinine, blood count, blood glucose and ultra- sensitive TSH are normal except for hypomagnesemia at 0.50 mmol. She is prescribed six tablets a day of magnesium for a week, and atenolol 25 mg per day. Driving is not recommended. A control of her metabolic panel, realized on November 3, 2011 is normal.

**Figure 1:**
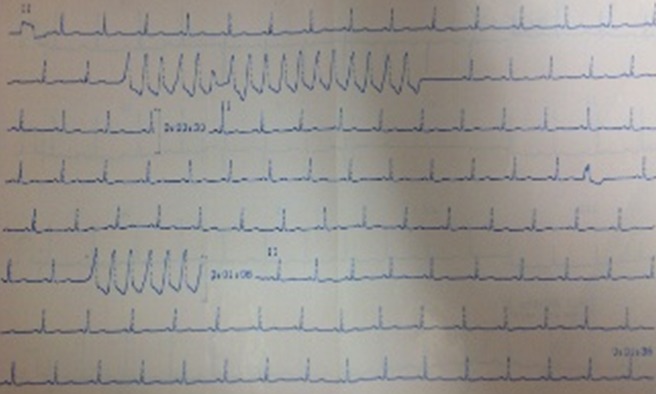
Electrocardiogram of a 4 month pregnant woman showing two unsustained ventricular tachycardia attack on lead II

Her lipothymia symptoms reoccurred on November 22, 2011, after a three weeks amendment. A 24-hours Holter ECG performed on November 23, 2011 is in sinus rhythm at 96 bpm, revealed many ventricular extrasystoles with R/T phenomenon. These extrasystoles included 30 doublets and six attacks of unsustained ventricular tachycardia (VT). She is prescribed amiodarone 200 mg daily which is quickly replaced by atenolol 50 mg and 100 mg causing the symptoms to subside. On January 25^th^, 2012, she reported chest tightness lasting less than a minute without palpitations or lipothymia. The patient is two months pregnant. Cardiac examination is normal. Troponin is negative and lipid profile is normal. Atenolol treatment is continued at 100 mg/day. On March 26^th^, 2012, Mrs. AMS is four months pregnant and is admitted to the emergency room for lipothymia, discomfort, and dizziness preceded by palpitations. She is anxious. Cardiac examination and blood pressure are normal. Pregnancy has a normal course. A surface ECG (Figure 1) records a monomorphic VT attack such as a left conduction slowness that was unsustained but rapid. Thyroid hormones, blood electrolytes, glucose, creatinine and blood count were normal. She was hospitalized in intensive care under ECG monitoring through a scope that recorded many unsustained VT attacks. Her treatment consisted potassium chloride, magnesium sulfate through an electric pump, the substitution of 100 mg atenolol by 200 mg acebutolol, and enoxaparin for primary prevention of venous thromboembolic disease.

**Figure 2:**
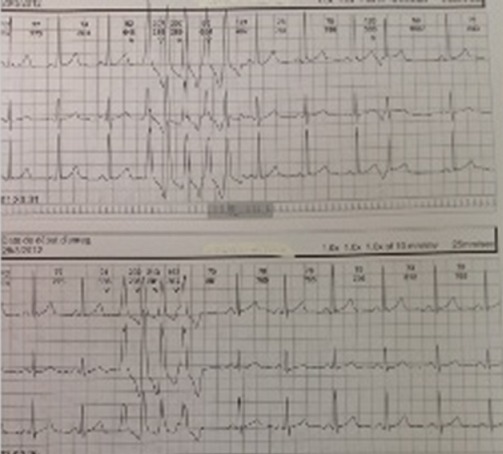
Unsustained ventricular tachycardia on a 24-hours Holter ECG tracing of a patient during pregnancy

Ventricular tachycardia attacks persisted under this treatment. Replacement of acebutolol by nadolol brought her back to normal sinus rhythm and she was discharged on the third day of hospitalization. An outpatient Holter ECG was performed on 03/28/2012 recorded ventricular extrasystoles numerous unsustained VT attacks (Figure 2) and rare supraventricular extrasystoles. She is readmitted on March 30^th^, 2012, for the same symptoms. A Holter ECG revealed frequent monomorphic ventricular extrasystoles (1287) of which 318 unsustained VT attacks. The combination of 100 mg flecainide (1/2 tablet three times per day) with 80 mg nadolol (1/4 tablet twice à day) controlled the arrhythmia and she was discharged on the tenth day of hospitalization. Under this treatment and oral solution of magnesium sulfate a Holter ECG monitoring revealed a marked improvement in monomorphic ventricular extrasystoles with only three doublets and one unsustained ventricular attack. Mrs. AMS is then transferred to another country where advanced technology electrophysiological study confirmed the infundibular type of her VT. She is successfully treated with radiofrequency ablation. Antiarrhythmic drugs are discontinued. At 27 months post-ablation Ms. AMS is doing well. She has had a vaginal delivery of a full term healthy child with no cardiac complication before undergoing radiofrequency.

## Discussion

Cardiac arrhythmias can occur during pregnancy in the presence or absence of an underlying organic heart disease [1, 2]. They are most often made of ventricular or supraventricular extrasystoles with a good prognosis. The mechanisms of these arrhythmias include hemodynamic changes during pregnancy, the main ones being an increase in total blood volume, an increase in heart rate inducing an increase in cardiac output and the decrease in peripheral arterial resistance [1] but also the intrinsic severity an underlying heart disease. The increase in heart rate is related to sympathetic stimulation, stress, pregnancy, hormonal phenomena and could generate cardiac arrhythmias [3].

Right ventricular outflow tract tachycardia on its own is a rhythm disturbance occurring on hearts that are usually healthy and it has good prognosis [3]. It is not specific to pregnancy and is most often seen in young women. Is the association with pregnancy a coincidence? This assumption is plausible in our case since the patient presented similar symptoms three months prior to the diagnosis of arrhythmias and prior to pregnancy. As a matter of fact, the recurrence of symptoms led to her outpatient visit to our hospital. It is known that ventricular tachycardia may be revealed or worsened by pregnancy [1, 4]. Although the association is fortuitous, the severity of the right ventricular outflow tract tachycardia remains a key question. Indeed several hospitalizations were required with a relative resistance to antiarrhythmic drugs (atenolol alone, acebutolol). The causes of ventricular tachycardia during pregnancy are multiple [1, 4–7] including peripartum cardiomyopathy, congenital heart disease that are operated on or unknown, and other types of heart diseases. In the case of the right ventricular outflow tract tachycardia, it is often a VT on a healthy heart. In our case, the absence of echocardiographic abnormalities, the absence of abnormal QT and a normal QRS morphology caused us to suggest right ventricular outflow tract tachycardia. Although the prognosis is generally benign, the persistent symptoms despite numerous medications, the heart rate that was accelerated at times, the psychological impact and the history of premature death of the father led us to want to rule out any unknown cause. Electrophysiological studies were not possible in a resource-limited setting. The effective combination therapy has been the association nadolol 80 mg and flecainide 150 mg. Ultimately radiofrequency performed after medical transfer allowed proper diagnosis and ideal therapy. The course of the disease brought satisfaction and a happy outcome for the pregnancy and the mother despite rhythmic storms. This is the classic trend of the association “ventricular tachycardia on a healthy heart-pregnancy”. Treatment is made of medical antiarrhythmic such as beta-blockers, verapamil, procainamide, and flecainide in the vast majority of cases [5, 8–12]. Ajmaline, procainamide or lidocaine are used in well tolerated cases but external electrical cardioversion is used in case of hemodynamic instability [12, 13]. Exceptionally the implantable automatic defibrillator is indicated for secondary prevention of sudden death in the presence of syncope or resuscitation due to ventricular tachycardia, ventricular fibrillation or ventricular flutter [13]. In our case, a resource-limited context, unpleasant symptoms for the patient, very rapid ventricular rhythms at times, have led us to transfer our patient for electrophysiological tests, then radiofrequency ablation. The postoperative course was uneventful and the patient remained asymptomatic 27 months after without antiarrhythmic drugs.

## Conclusion

This case brings forth the problematic issue of tachycardia associated with pregnancy and specifically the issue of optimal management of conventional drugs resistant cases through interventional cardiology in developing countries.
